# Topological Modification of Brain Networks Organization in Children With High Intelligence Quotient: A Resting-State fMRI Study

**DOI:** 10.3389/fnhum.2019.00241

**Published:** 2019-07-12

**Authors:** Ilaria Suprano, Chantal Delon-Martin, Gabriel Kocevar, Claudio Stamile, Salem Hannoun, Sophie Achard, Amanpreet Badhwar, Pierre Fourneret, Olivier Revol, Fanny Nusbaum, Dominique Sappey-Marinier

**Affiliations:** ^1^Univ. Lyon, INSA-Lyon, Université Claude Bernard Lyon 1, UJM-Saint Étienne, CNRS, INSERM, CREATIS UMR 5220, Lyon, France; ^2^Univ. Grenoble Alpes, INSERM, U1216, Grenoble Institut Neurosciences, Grenoble, France; ^3^Nehme and Therese Tohme Multiple Sclerosis Center, Faculty of Medicine, American University of Beirut, Beirut, Lebanon; ^4^GIPSA-Lab, UMR CNRS 5216, Université Grenoble Alpes, Grenoble, France; ^5^Centre de Recherche de l’Institut Universitaire de Gériatrie de Montréal, Université de Montréal, Montreal, QC, Canada; ^6^Service de Psychopathologie du Développement de l’Enfant et de l’Adolescent, Hospices Civils de Lyon, Lyon, France; ^7^Laboratoire Parcours Santé Systémique (Equipe d’Accueil 4129), Université de Lyon, Université Claude Bernard-Lyon 1, Lyon, France; ^8^Centre PSYRENE, Lyon, France; ^9^CERMEP – Imagerie du Vivant, Université de Lyon, Lyon, France

**Keywords:** intelligence, functional MRI, resting state, functional connectivity, brain networks, hub disruption index, children

## Abstract

The idea that intelligence is embedded not only in a single brain network, but instead in a complex, well-optimized system of complementary networks, has led to the development of whole brain network analysis. Using graph theory to analyze resting-state functional MRI data, we investigated the brain graph networks (or brain networks) of high intelligence quotient (HIQ) children. To this end, we computed the “hub disruption index κ,” an index sensitive to graph network modifications. We found significant topological differences in the integration and segregation properties of brain networks in HIQ compared to standard IQ children, not only for the whole brain graph, but also for each hemispheric graph, and for the homotopic connectivity. Moreover, two profiles of HIQ children, homogenous and heterogeneous, based on the differences between the two main IQ subscales [verbal comprehension index (VCI) and perceptual reasoning index (PRI)], were compared. Brain network changes were more pronounced in the heterogeneous than in the homogeneous HIQ subgroups. Finally, we found significant correlations between the graph networks’ changes and the full-scale IQ (FSIQ), as well as the subscales VCI and PRI. Specifically, the higher the FSIQ the greater was the brain organization modification in the whole brain, the left hemisphere, and the homotopic connectivity. These results shed new light on the relation between functional connectivity topology and high intelligence, as well as on different intelligence profiles.

## Introduction

Since the introduction of the “general model” described by Spearman in 1904 (Spearman, 1904), and the first standardized psychological tests developed by Binet (1905), the concept of intelligence has been a challenge in cognitive research (Haier, 2016). At present, neuropsychological tests, such as the Wechsler Intelligent scale for Children (WISC) (Wechsler, 2003), can provide a reliable estimation of the intelligence quotient (IQ), and help clinically assess children with high intelligence quotient (HIQ). HIQ children show better cognitive abilities in reasoning, problem solving, memory, language, visuospatial perception, and learning (Vaivre-Douret, 2011). These abilities are usually revealed by the four WISC subscales: the verbal comprehension index (VCI), the perceptual reasoning index (PRI), the working memory index (WMI), and the processing speed index (PSI), all of which provide a measurement of the full-scale IQ (FSIQ) (Berk, 1982). However, some HIQ children may present associative learning troubles, attention deficits, and emotional and social maladjustments – described as the “di-synchrony syndrome” (Silverman, 1997; Terrassier, 2009) and usually detected as a significant difference between VCI and PRI values. These clinical and neuropsychological observations have led us to define two profiles of HIQ children: the homogeneous HIQ (Hom-HIQ) and the heterogeneous HIQ (Het-HIQ), the latter characterized by a significant difference between VCI and PRI.

Different brain functions have been investigated in intellectually gifted children and adolescents using task-based functional MRI (fMRI). Increased activations in correlation with general intelligence have been reported in the fronto-parietal network (FPN), the bilateral posterior–parietal regions (Lee et al., 2006), as well as in the prefrontal–dorsolateral regions associated with reasoning (Noveck et al., 2004) and memory (Gray et al., 2003). However, neural efficiency may vary as a function of task difficulty (Neubauer and Fink, 2009). These findings led Jung and Haier (2007) to propose the parieto-frontal integration theory (P-FIT), which posits that intelligence is correlated with the connectivity between parietal and frontal as well as temporal regions with a predominance in the left hemisphere. This model was recently updated and extended to encompass other cerebral regions and subcortical structures (Basten et al., 2015).

Resting-state fMRI (rs-fMRI) allows the measurement of functional connectivity (FC) in large-scale brain networks dedicated either to specific cognitive processing demands (Shirer et al., 2012) or to intrinsic brain activity (Fox et al., 2005). Based on these approaches, Sherman et al. (2016) reported a correlation with intelligence in the default mode network (DMN) and the central executive network (CEN) of early adolescents. In a cohort of young children, Langeslag et al. (2013) reported associations between high nonverbal intelligence and increased FC between parietal and frontal, and parietal and anterior cingulate regions. Based on an exploratory mapping of the literature, and a network analysis of the Human Connectome Project (HCP) data, Hearne et al. (2016) showed that both the DMN and the FPN were strongly correlated with high intelligence scores in young adults. Moreover, the cognitive functions related to adult intelligence (measured using IQ) seemed to correlate with the FC of homotopic regions, which was reported reduced in the primary sensorimotor cortex (Santarnecchi et al., 2015). Exploration of homotopic connectivity is currently gaining interest, as it has been demonstrated to robustly increase with advancing gestational age in the fetus (Thomason et al., 2013), and be highly consistent within and across subjects (Finn et al., 2015).

The concept of intelligence being embedded not only in a single brain network, but rather in a complex organization of communicating brain networks has recently emerged (Ponsoda et al., 2016). Graph theory (Watts and Strogatz, 1998) is particularly relevant for modeling brain FC as a global efficient network, supporting both segregated and distributed information processing (Sporns and Zwi, 2004) that is modeled by a “small-world” topology (Achard and Bullmore, 2007). This approach was recently applied to characterize the neuronal substrate of intelligence attributable to both structural and FC, using diffusion tensor imaging (DTI) (Kim et al., 2016; Kocevar et al., 2019) and rs-fMRI (Van den Heuvel et al., 2009; Hilger et al., 2017a; Kruschwitz et al., 2018). These studies investigated specific metrics reflecting network integration properties [global efficiency (GE) and degree (D)], segregation properties [local efficiency (LE) and clustering coefficient (CC)], and hubness properties [betweenness centrality (BC)]. Van den Heuvel et al. (2009) reported a significant correlation between GE and intelligence, though this finding was not reproduced in the larger HCP cohort (Kruschwitz et al., 2018). Furthermore, LE was demonstrated to be positively correlated with FSIQ in regions of the salience network and negatively in the temporo-parietal junction (Hilger et al., 2017a). Taken together, these results suggest a possible HIQ-related global modification of network topology. This was recently conceptualized by Barbey (2018) who suggested that “intelligence depends on the dynamic organization of brain networks, modifying their topology and community structure in the service of system-wide flexibility and adaptation.” Therefore, we propose to investigate the relationship between intelligence and brain network FC using a graph organization measure, the “hub disruption index κ.” This approach has been applied to several brain pathologies demonstrating significant brain network reorganizations, such as coma (Achard et al., 2012), epilepsy (Gendon et al., 2015), and stroke (Termenon et al., 2016a). Moreover, the hub disruption index has been shown to be more reliable and sensitive than global graph metrics to detect group differences between patients and healthy controls (Termenon et al., 2016b).

In this work, we assessed the topological modification of brain networks organization in HIQ children using the κ index to characterize the neural substrate of intelligence. Graph analyses were performed in different networks: the whole brain, both cerebral hemispheres (given the asymmetry of brain functions), and between homotopic regions. Whether FC changes relate to FSIQ and/or to its subscales was also investigated by correlational analysis. This approach will allow for better understanding of the differences in FC substrate between high and standard IQ children, as well as between the two HIQ profiles.

## Materials and Methods

### Participants

Fifty-eight children (44 males and 14 females) ages 8–12 (mean age 10.1 ± 1.2) years were recruited from the children psychiatry department of Lyon’s Neurological Hospital, the PSYRENE Center, a psychological center for high IQ children and adults, and via advertisement in schools for controls. Children with neurological diseases, learning disabilities, and psychotropic treatments were excluded from this study. Children underwent the fourth edition of WISC (WISC-IV) test and their FSIQ was established from the results of its four subscales (VCI, PRI, WMI, and PSI). Children with a high Intelligence Quotient (FSIQ > 130 or VCI > 130) were labeled as HIQ children and two HIQ profiles were defined based on score difference between VCI and PRI (Table 1). This prospective study was approved by the local ethics committee (CPP Sud-Est IV) and the French National Agency for Medicine and Health Products Safety (ANSM). Written informed consent was obtained from the parents of all participants.

**Table 1:** Population characteristics (mean ± SD): age, full-scale IQ (FSIQ), verbal comprehension index (VCI), perceptual reasoning index (PRI), processing speed index (PSI), and working memory index (WMI) for standard intelligence quotient (SIQ), high intelligence quotient (HIQ) groups, and HIQ subgroups: homogeneous (Hom-HIQ) and heterogeneous (Het-HIQ).

	SIQ (*n* = 12)	HIQ (*n* = 37)	Hom-HIQ (*n* = 15)	Het-HIQ (*n* = 22)
Age	10.0 ± 1.1	10.0 ± 1.1	9.8 ± 0.8	10.1 ± 1.3
FSIQ	104.1 ± 8.5	134.1 ± 12.3^∗∗∗^	141.6 ± 11.8	129.3 ± 10.2^#^
VCI	108.1 ± 6.8	143.0 ± 9.8^∗∗∗^	141.6 ± 12.5	143.9 ± 8.0
PRI	97.7 ± 6.9	124.1 ± 13.8^∗∗∗^	136.4 ± 9.2	116.8 ± 10.5^##^
WMI	95.6 ± 10.3	117.2 ± 15.3^∗∗∗^	125.3 ± 15.5	112.0 ± 13.0^#^
PSI	102.3 ± 15.1	107.8 ± 16.8	114.3 ± 18.4	103.8 ± 14.9

*^∗∗∗^p < 0.001 when comparing HIQ and SIQ using a Wilcoxon test. ^##^p < 0.01; ^###^p < 0.001 when comparing Hom-HIQ and Het-HIQ using Dunn’s post hoc test after the comparison of SIQ, Hom-HIQ, and Het-HIQ using Kruskal–Wallis test.*

### MRI Acquisitions

Magnetic resonance imaging (MRI) examinations were performed on a 1.5T Siemens Sonata MRI system (Erlangen, Germany) with an eight-channel head-coil at the MRI Department of CERMEP – Imagerie du Vivant. A structural 3D T1-weighted MPRAGE sequence was first acquired in the sagittal plane with a 1-mm isotropic spatial resolution (TI/TE/TR = 1100/3.93/1970 ms, FOV: 256 × 256 × 176 mm, 8 min acquisition duration). Then a full examination with task fMRI, DTI, and rs-fMRI was conducted. rs-fMRI data were recorded using an EPI BOLD sequence (250 scans, TR = 2500 ms, TE = 50 ms, voxel size = 3.4 × 3.4 × 3 mm) while subjects lay quietly at rest with eyes open and fixating on a projected cross for 10.3 min. For this study only the rs-fMRI (at the end of the exam) is reported.

### Data Preprocessing

The rs-fMRI data were preprocessed using SPM12 software^1^. For each subject, functional images were corrected for delay between slice acquisitions, motion, and co-registered to the anatomical image. Time series data were not spatially smoothed because the smoothing step introduces spurious spatial correlations between adjacent regions (Fornito et al., 2010). Using Art toolbox that evaluates the scans affected by scan-to-scan motion, we scrubbed the data carefully. When scans with movements >3 mm or with an exceptionally high variation (signal >4 standard deviations from mean) were found, a corresponding artifact regressor was constructed. A participant’s data were excluded if >20% of the available scans were affected by head motion. Motion parameters for each group were measured and compared among the groups to verify that the correlation results were not influenced by inter-group motion differences (Power et al., 2012). The anatomical MRI from each participant was segmented into six different brain and non-brain tissues according to prior tissue probability maps. This step generates gray matter (GM), white matter (WM), and cerebrospinal fluid (CSF) probability maps that will be eventually be used to extract time series to compute the graph. These maps were further normalized to the MNI152 template using DARTEL, a diffeomorphic registration method that accurately align brains within the MNI space (Ashburner, 2007). This registration provides a deformation field that was then applied to functional and anatomical images to be later used to extract the time series to compute the graphs. The structural images were parcellated into 84 cortical, subcortical, and cerebellar areas according to the Desikan atlas (Desikan et al., 2006). Regional mean time series were estimated by averaging the fMRI time series over all voxels in each parcel weighted by GM probability map using the Conn Toolbox^2^. Finally, time series were regressed by the residual contamination from WM, CSF signals, motion parameters, and outliers detected using the ART toolbox and band-pass filtering was applied using wavelets transforms.

### Wavelets Decomposition

Following the approach proposed by Achard et al. (2006), time series were decomposed using dyadic wavelet transforms that partitions the total energy of a signal over a set of compactly supported basis functions, each of which is uniquely scaled in frequency and located in time. The pairwise interregional correlations between wavelets coefficients of fMRI time series extracted from each individual data set were estimated for four wavelet scales. Because frequencies below 0.1 Hz contain relevant information in rs-fMRI, we restricted our analysis to two wavelet scales: the scale 2 from *f* = 1/(8 TR) to *f* = 1/(4 TR), that represents the frequency interval 0.05–0.1 Hz, and the scale 3 from *f* = 1/(16 TR) to *f* = 1/(8 TR), that represents the frequency interval 0.025–0.05 Hz. At this stage, we measured the percentage of significant correlations obtained from the two scales. Since the wavelet scale 2 presents a higher percentage of significant correlations, we chose to proceed with the graph computation using the correlation matrices of this scale.

### Graph Construction

To construct the binarized graph, two steps are necessary. Following Alexander-Bloch et al. (2012), we kept the graph fully connected, using the minimum spanning tree based on the absolute correlation matrix. This creates a preliminary graph that contains a number of edges equal to the number of nodes minus one. In the second step, the remaining absolute values of the correlation matrices were thresholded to create an adjacency matrix that defines, for each subject, an unweighted and undirected graph using two graph costs: 15 and 20%. The choice of these values was guided by the results of a test–retest study of graph metrics derived from graph analysis of rs-fMRI dataset, where the cost of about 20% provides a good reliability of all graph metrics (Termenon et al., 2016b). Since our results obtained with the two costs were concordant, we reported in this study those related to 15%, allowing a larger inclusion of subjects and a greater robustness of the correlations. After having verified that the small-world property was satisfied for each subject and that the topology of the three groups was the same, four topological metrics were estimated for each node using Brain Connectivity Toolbox^3^: D, BC, LE, and CC. When the graph was computed with the 84 regions from the Desikan atlas, we referred to it as the “whole brain networks” in the manuscript. When the graph was computed with the 42 regions of the right (or left) hemisphere, we referred to it as the “right hemispheric networks” (or the “left hemispheric networks”). Finally, we considered the connectivity related to the homotopic regions: 42 nodes in each hemisphere as the “homotopic connectivity.”

### Hub Disruption Index (κ) Estimation

**FIGURE 1:**
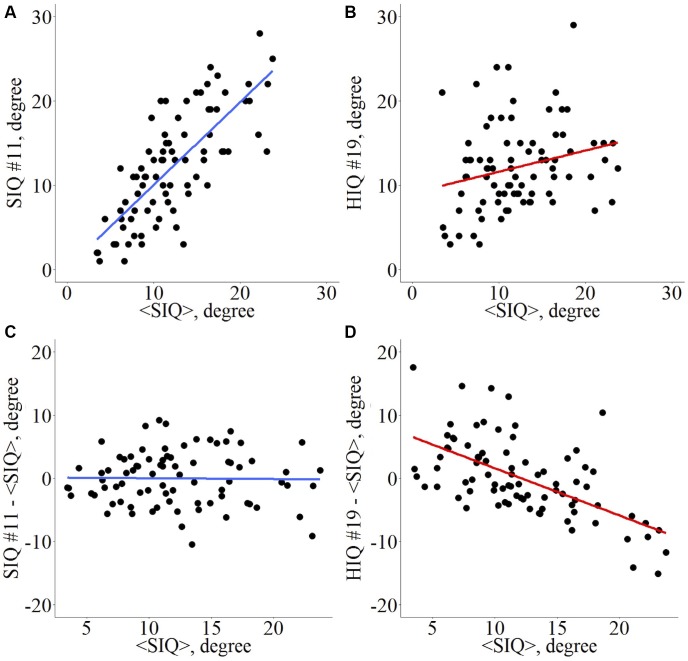
Hub disruption index κ computation for a graph metric. Given a set of nodes from an atlas *N_i_*, *i* ∈ [1, *n*], and a nodal metric *M*, each node *i* presents a value *M_i_*, *i* ∈ [1, *n*] for a given subject. Across a set of reference subjects *R_j_*, *j* ∈ [1, *m*], the averaged nodal metric can be computed <*M_i_*>*_R_*. For each individual *I_k_*, *k* ∈ [1, *p*], whatever its status (patient, HIQ child, or healthy subject), its metric in each node is *M_i,I_k__* and the difference in nodal metric with the reference group is *M_i,I_k__* – <*M_i_*>*_R_*. The scatterplot with all nodes is computed with <*M_i_*>*_R_* in abscissa and *M_i,I_k__* – <*M_i_*>*_R_* in ordinates. For each node *i*, if the nodal metric is close to the reference’s nodal metric, then the linear trend of this plot is about 0. Conversely, if the nodal metrics are reduced in some nodes and increased in others, then the linear trend will differ from 0. κ is the slope of the regression line computed on this scatter plot. Example for the nodal degree (D) as metric of interest. *D_i,I_* vs. <*D_i_*>*_R_* for a standard intelligence quotient (SIQ) child **(A)** and for a high intelligence quotient (HIQ) child **(B)**, *D_i,I_* – <*D_i_*>*_R_* vs. <*D_i_*>*_R_* for a SIQ child **(C)** is scattered around a horizontal line (κ ∼ 0), whereas for a HIQ child **(D)** is scattered around a negatively sloping line (κ < 0).

Introduced by Achard et al. (2012), the “Hub Disruption Index” describes the topological changes of an individual subject brain networks with respect to a referential networks topology from a group of reference subjects. To understand how this index is defined, consider a nodal metric, for example the degree (D), and plot the D value of each node for a SIQ subject against the average D values of the corresponding nodes in the SIQ group (Figure 1A). Since for a SIQ subject the nodal metric values are close to the average value for the same node computed in the SIQ group, the distribution of the points falls approximately on a positive slope line (*y* = *x*). Constructing the same plot for a HIQ subject, we can observe that the point cloud does not scatter around the same slope (Figure 1B), so they are not well predicted by the SIQ average D. For each nodal metric κ is defined following several steps. The SIQ group mean metric of each node was first subtracted from the metric of the corresponding node in an individual subject. This difference was further plotted against the SIQ group mean for all the nodes and the gradient of the linear regression that models this points cloud represents κ. According to this definition, data of a SIQ subject will scatter around a horizontal line (κ ∼ 0) (Figure 1C), while for a HIQ subject data will follow a negative slope (κ < 0) (Figure 1D). κ index was calculated for D, BC, LE, and CC in whole brain networks (κ_D_, κ_BC_, κ_LE_, and κ_CC_) and in both left ($\kappa_{\text{D}}^{\text{L}}$,$\kappa_{\text{BC}}^{\text{L}}$,$\kappa_{\text{LE}}^{\text{L}}$, and $\kappa_{\text{CC}}^{\text{L}}$) and right ($\kappa_{\text{D}}^{\text{R}}$,$\kappa_{\text{BC}}^{\text{R}}$,$\kappa_{\text{LE}}^{\text{R}}$, and $\kappa_{\text{CC}}^{\text{R}}$) networks, and for functional homotopic connectivity (κ^HC^).

### Statistical Analysis

First, statistical differences between κ indices of each group were computed using permutation tests, by randomly reassigning subjects to three groups: 12 children played the role of the SIQ group, 15 of Hom-HIQ group, and 22 of Het-HIQ group. For each subject, κ was computed following its definition. This process was repeated for 1000 permutations of the data to sample the null distribution of κ. The *p*-value was computed counting how many times the κ-values were higher than the one obtained using the true SIQ and HIQ groups. As the κ definition is based on the reference group (SIQ), its homogeneity was controlled using the Grubbs’ test. One outlier was identified and excluded from the SIQ group. Furthermore, as nodes could play different roles in brain networks organization, we tested for metric differences in graph’s nodes between each HIQ group and SIQ. A statistical analysis was performed at each node using a non-parametric Wilcoxon test and a Benjamini–Hochberg correction for multiple comparisons.

Second, the correlations between intelligence scores (FSIQ, VCI, PRI) and hub disruption index (κ) were analyzed using a non-parametric Spearman correlation coefficient (ρ) controlling for sex. Correlation significance level was evaluated replicating a permutation test, by randomly reassigning WISC-IV scores to the subjects, for 1000 iterations. The *p*-value was computed counting how many times the ρ-values were higher than the one obtained with our true intelligence scores and corrected for multiple comparisons using the Benjamini–Hochberg correction.

All statistical analyses were computed on R^4^ and since the groups were matched in age, with small standard deviation values. No regression of age effect was applied.

## Results

### Modification of FC Organization With Intelligence

In the whole brain networks, HIQ children showed significant differences in hub disruption indices of several graph metrics, namely κ_D_ (*p* < 0.01), κ_CC_ (*p* < 0.05), and κ_LE_ (*p* < 0.05), compared to SIQ children (Table 2A). These results suggest significant topological modifications in the graph’s integration and segregation properties. Locally, D was significantly decreased in the left dorsolateral prefrontal cortex (BA 9-10-46) (*p* < 0.01), indicating decreased prefrontal FC in HIQ children. These networks changes were further assessed in the two HIQ subgroups separately, relative to the SIQ group. The Hom-HIQ group didn’t show any significant changes, whereas the Het-HIQ group showed significant changes in integration and segregation properties related to all graph metrics (Table 2A). A significant D reduction was also locally observed in the left dorsolateral prefrontal node (*p* < 0.01) of the Het-HIQ group, while a trend toward a decreased D was measured in the left inferior parietal cortex (*p* < 0.1).

**Table 2:** Hub disruption indices (κ) in HIQ, homogeneous HIQ (Hom-HIQ), and heterogeneous HIQ (Het-HIQ) groups measured in whole brain networks (A), left and right hemispheres networks (B and C), and between homotopic regions (D).

Networks	κ	HIQ (*n* = 37)	Hom-HIQ (*n* = 15)	Het-HIQ (*n* = 22)
(A) Whole brain	κ_BC_	−0.472	−0.475	−0.470
	κ_D_	−0.317^∗∗^	−0.228	−0.378^∗∗^
	κ_LE_	−0.291^∗^	−0.201	−0.352^∗^
	κ_CC_	−0.350^∗^	−0.259	−0.413^∗^
(B) Left hemisphere	$\kappa_{\text{BC}}^{\text{L}}$	−0.399	−0.396	−0.402
	$\kappa_{\text{D}}^{\text{L}}$	−0.299^∗∗^	−0.306^∗^	−0.294^∗∗^
	$\kappa_{\text{LE}}^{\text{L}}$	−0.340	−0.299	−0.369
	$\kappa_{\text{CC}}^{\text{L}}$	−0.399	−0.342	−0.438
(C) Right hemisphere	$\kappa_{\text{BC}}^{\text{R}}$	−0.311	−0.292	−0.324
	$\kappa_{\text{D}}^{\text{R}}$	−0.228	−0.070	−0.336^∗^
	$\kappa_{\text{LE}}^{\text{R}}$	−0.364	−0.209	−0.471^∗^
	$\kappa_{\text{CC}}^{\text{R}}$	−0.406	−0.264	−0.502
(D) Homotopic	κ^HC^	−0.198^∗^	−0.214^∗^	−0.187^∗^

*^∗^p < 0.05; ^∗∗^p < 0.01; when testing significance of κ-values in HIQ, Hom-HIQ, or Het-HIQ groups compared to SIQ group using permutation test (number of permutations = 1000).*

Since the neural substrate of high intelligence may be related to hemispheric characteristics (Hearne et al., 2016; Nusbaum et al., 2017), we additionally explored the topological changes of brain FC organization by computing the κ values of intra-hemispheric networks connectivity (ignoring inter-hemispheric connectivity). In the left hemisphere, only integration properties measured by κ_D_ (*p* < 0.01) were significantly reorganized in the HIQ group, compared to the SIQ group (Table 2B). Specifically, nodal analysis showed a significant D reduction (*p* < 0.01) in the left dorsolateral prefrontal node (BA 9-10-46). No significant changes were found in the right hemisphere (Table 2C). When exploring the intra-hemisphere graph networks of each HIQ subgroup, significant modifications of integration properties were found in the left hemisphere of both Hom-HIQ (*p* < 0.05) and Het-HIQ groups (*p* < 0.01), relative to SIQ (Table 2B). This difference was locally highlighted by a D reduction in the left dorsolateral prefrontal cortex of the Het-HIQ group. While right hemisphere networks were not significantly modified in the Hom-HIQ group, integration and segregation properties, measured by D (*p* < 0.05) and LE (*p* < 0.05), were significantly changed in the Het-HIQ group (Tables 2C, 3). In sum, both HIQ groups showed modifications of integration properties in the left hemisphere, while only the Het-HIQ profile showed changes of integration and segregation properties in the right hemisphere.

**Table 3:** Coefficients of non-parametric correlations (ρ) between the hub disruption index (κ) of different nodal metrics [betweenness centrality (BC), degree (D), local efficiency (LE), clustering coefficient (CC), and homotopic connectivity (HC)] with intelligence scores [FSIQ, VCI, and PRI] at different network levels: whole brain (A), left and right hemispheres (B and C), and homotopic regions (D).

Networks	κ	FSIQ	VCI	PRI
(A) Whole brain	κ_BC_	−0.344^∗^	−0.316^∗^	−0.306^∗^
	κ_D_	−0.277^∗^	−0.310^∗^	−0.218
	κ_LE_	−0.153	−0.250^∗^	−0.138
	κ_CC_	−0.133	−0.253^∗^	−0.131
(B) Left hemisphere	$\kappa_{\text{BC}}^{\text{L}}$	−0.250	−0.138	−0.107
	$\kappa_{\text{D}}^{\text{L}}$	−0.267^∗^	−0.213	−0.295^∗^
	$\kappa_{\text{LE}}^{\text{L}}$	−0.290^∗^	−0.305^∗^	−0.255^∗^
	$\kappa_{\text{CC}}^{\text{L}}$	−0.250	−0.279^∗^	−0.228
(C) Right hemisphere	$\kappa_{\text{BC}}^{\text{R}}$	−0.143	−0.171	−0.100
	$\kappa_{\text{D}}^{\text{R}}$	−0.172	−0.223	−0.028
	$\kappa_{\text{LE}}^{\text{R}}$	−0.209	−0.261^∗^	−0.115
	$\kappa_{\text{CC}}^{\text{R}}$	−0.226	−0.260^∗^	−0.135
(D) Homotopic	κ^HC^	−0.396^∗∗^	−0.431^∗∗^	−0.379^∗∗^

*^∗^p < 0.05; ^∗∗^p < 0.01 when testing significance level of correlations using permutation testing (number of permutations = 1000).*

Since homotopic FC has been shown to correlate with IQ (Santarnecchi et al., 2015), we analyzed the FC between homotopic regions in each HIQ group by measuring their κ indices. Significant changes (*p* < 0.05) in homotopic connectivity were found in the HIQ group compared to the SIQ group (Table 2D). Exploring the two HIQ subgroups separately, significant changes (*p* < 0.05) of the homotopic FC were found in both Hom-HIQ and Het-HIQ groups (Table 2D). At a regional level, a trend toward increased homotopic FC was found in the amygdala nodes of the Het-HIQ group (*p* < 0.1).

### Correlation Between FC Organization and Intelligence

We further investigated how these topological organization changes could be related to the high abilities of HIQ children. A correlation analysis between the previously measured κ indices and the different IQ scales was performed in different brain networks.

**FIGURE 2:**
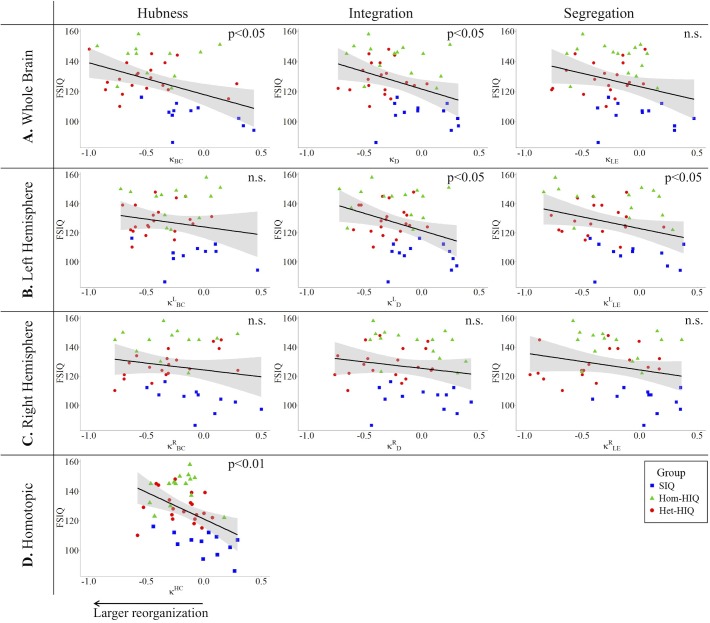
Correlations between full-scale intelligence quotient (FSIQ) and hub disruption indices (κ) of graph metrics measured in: **(A)** the whole brain networks, **(B)** the left hemisphere networks, **(C)** the right hemisphere networks, and **(D)** the homotopic nodes. Significant correlations were measured for κ describing hubs (κ_BC_) and integration properties (κ_D_) in the whole brain networks, for integration and segregation properties in the left hemisphere networks ($\kappa_{\text{D}}^{\text{L}}$ and $\kappa_{\text{LE}}^{\text{L}}$) and for homotopic connectivity (κ^HC^). No significant correlations were found in the right hemisphere networks.

**FIGURE 3:**
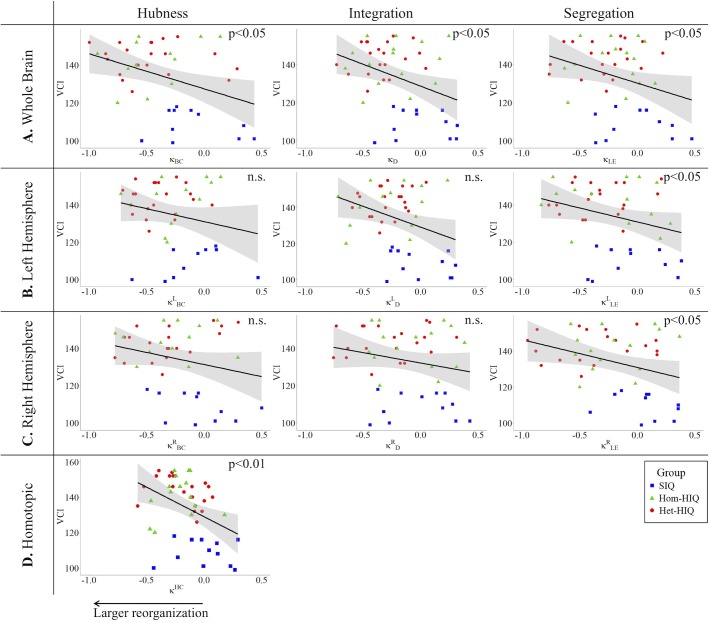
Correlations between verbal comprehension index (VCI) and hub disruption indices (κ) of graph metrics measured in: **(A)** the whole brain networks, **(B)** the left hemisphere networks, **(C)** the right hemisphere networks, and **(D)** the homotopical nodes. Significant correlations were measured for κ describing hubs properties in the whole brain networks (κ_BC_), for integration properties in the whole brain networks (κ_D_), and for segregation properties in the whole brain networks (κ_CC_ and κ_LE_), in the left ($\kappa_{\text{CC}}^{\text{L}}$ and $\kappa_{\text{LE}}^{\text{L}}$) and right hemisphere ($\kappa_{\text{CC}}^{\text{R}}$,$\kappa_{\text{LE}}^{\text{R}}$), and for homotopic connectivity (κ^HC^).

**FIGURE 4:**
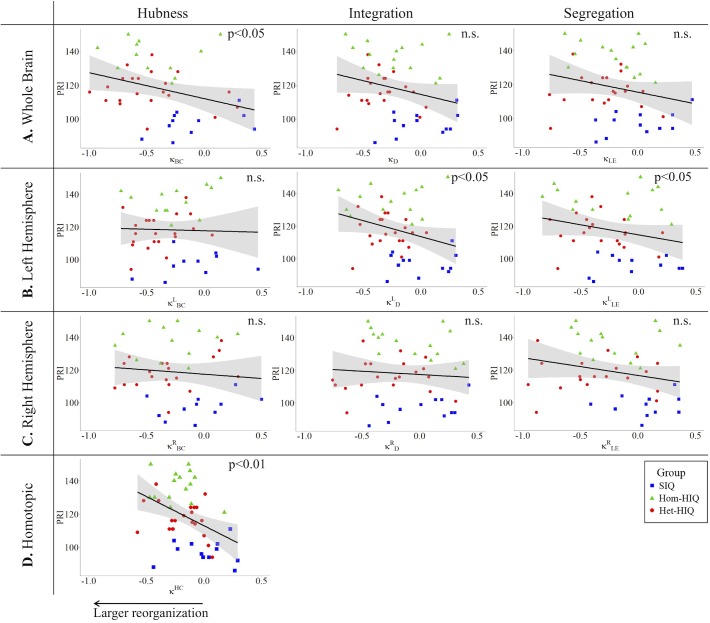
Correlations between perceptual reasoning index (PRI) and hub disruption indices (κ) of graph metrics measured in: **(A)** the whole brain networks, **(B)** the left hemispheric networks, **(C)** the right hemispheric networks, and **(D)** the homotopical nodes. Significant correlations were measured for κ describing hubs properties in the whole brain networks (κ_BC_), for integration ($\kappa_{\text{D}}^{\text{L}}$) and segregation properties in the left hemisphere ($\kappa_{\text{LE}}^{\text{L}}$), and for homotopic connectivity (κ^HC^).

**FIGURE 5:**
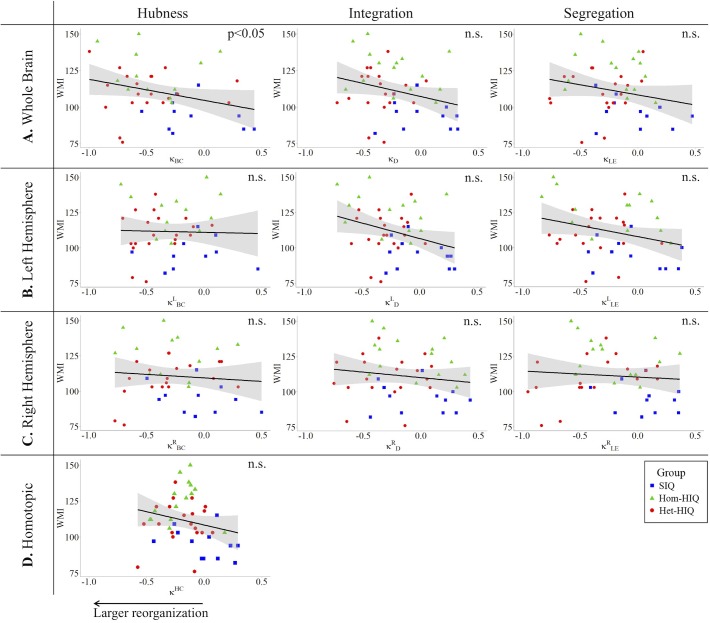
Correlations between working memory index (WMI) and hub disruption indices (κ) of graph metrics measured in: **(A)** the whole brain networks, **(B)** the left hemisphere networks, **(C)** the right hemisphere networks, and **(D)** the homotopical nodes. Significant correlations were measured for κ describing hubs properties in the whole brain networks (κ_BC_).

In the whole brain networks, significant negative correlations were found between the hub disruption indexes, related to integration (κ_D_) or hubness (κ_BC_) properties, and the FSIQ and VCI (Table 3A). As shown in Figure 2, the higher the FSIQ, the greater the hub disruption index, thereby reflecting a high sensitivity of κ_BC_ to highlight the differences in FSIQ. κ_BC_ was also correlated (*p* < 0.05) with PRI and WMI (Figure 4, 5). In addition, VCI significantly (*p* < 0.05) correlated with all the hub disruption indices (κ_BC_, κ_D_, κ_LE_, and κ_CC_) (Table 3A and Figure 3). When separately exploring the networks in the left hemisphere, FSIQ and PRI were negatively correlated with modifications of integration and segregation metrics ($\kappa_{\text{D}}^{\text{L}}$ and $\kappa_{\text{LE}}^{\text{L}}$) (*p* < 0.05), while VCI was correlated only with the modifications of segregation metrics ($\kappa_{\text{CC}}^{\text{L}}$ and $\kappa_{\text{LE}}^{\text{L}}$) (*p* < 0.05). In the right hemisphere, only the segregation metrics changes ($\kappa_{\text{CC}}^{\text{R}}$and $\kappa_{\text{LE}}^{\text{R}}$) were significantly correlated (*p* < 0.05) with VCI (Figure 3). Finally, the strongest correlations (*p* < 0.01) were observed between the hub disruption index in homotopic regions (κ^HC^) and the three major intelligence subscales, namely FSIQ, VCI, and PRI (Figure 3–5).

## Discussion

The hub disruption index κ was used in this study, on the one hand to uncover the topological organization modification of brain networks in children with high intelligence, and on the other hand, to investigate whether these changes could be related to their specific cognitive profiles.

### Brain Networks Changes With High Intelligence

Our study provided evidence that FC networks in HIQ children undergo modifications of integration and segregation properties, in comparison to SIQ children. Indeed, κ_D_ (related to integration properties) as well as κ_LE_ and κ_CC_ (related to networks segregation properties) were modified in the whole brain networks, while only κ_D_ was changed in the left hemisphere of HIQ children. This last result was observed in both subgroups of HIQ children, showing common integration properties changes in the left hemisphere. Conversely, in the right hemisphere, modifications of integration and segregation properties were only highlighted in the Het-HIQ subgroup. These changes in integration properties support the hypothesis that intelligence is based on better neural efficiency, which promotes better information transmission. Our results are in concordance with previous observations of greater FC associated with high intelligence in certain regions of the fronto-parietal and DMNs (Jung and Haier, 2007; Basten et al., 2015; Hearne et al., 2016), two nodes of the salience network, and one node of the DMN (Hilger et al., 2017a).

The WISC-IV test provides a global index (FSIQ), as well as four subscales including verbal (VCI) and non-verbal (PRI) indices, that are sensitive to different cognitive capabilities. Based on a significant difference between these two subscales, two profiles of HIQ children were identified, namely the Hom-HIQ and the Het-HIQ (Table 1). It is interesting to underline that we found a significant modification of brain networks organization common to both Hom-HIQ and Het-HIQ subgroups. Additionally, we also observed specific changes in the Het-HIQ subgroup. These results support the existence of different intelligence profiles that should be taken into account during investigations on intelligence. Moreover, our results showed that high intelligence-associated functional neural changes occur differently in the left and right hemispheres. Surprisingly, the neuroimaging literature does not report such lateralization, except for a rs-fMRI study by Santarnecchi et al. (2015), a DTI study by Tamnes et al. (2010), and our previous DTI study of Hom-HIQ and Het-HIQ children that included subjects described in the present study (Nusbaum et al., 2017). This last study found increased structural connectivity (measured using axial diffusivity) in both Hom- and Het-HIQ groups, with the Het-HIQ group being more lateralized in the left hemisphere and the Hom-HIQ group in the right. These findings demonstrated that brain lateralization of both structural and FC play a significant role in intelligence. This observation led us to further investigate the role of homotopic regions in intelligence. During brain development, homotopic FC was shown to increase with advancing gestational age (Thomason et al., 2013). Along the lifespan, sensorimotor regions tend to show increasing homotopic FC, whereas prefrontal higher-order processing regions show decreasing connectivity (Zuo et al., 2010). Santarnecchi et al. (2015) addressed the relation between homotopic connectivity and intelligence in adults. Reduced homotopic connectivity was reported in above average-IQ versus average-IQ subjects in the primary sensory regions, suggesting that a downgrading of inter-hemispheric transmission at rest could be associated with higher intelligence for efficiency purpose. In our study, significant changes of the homotopic connectivity were found in HIQ children and in both HIQ subgroups, reflecting decreased connectivity in some node pairs and an increase in others.

### Correlation Between Brain Networks Changes and IQ Subscales

We additionally demonstrated that the reported topological organization changes were correlated with cognitive abilities, thus supporting the hypothesis that intelligence relates to the brain networks functional organization (Table 3). As illustrated in Figure 1, FSIQ significantly correlated with the hub’s changes, as measured by κ_BC_ in the whole brain networks. Hubs’ modifications, therefore, occur in children with high cognitive abilities, as demonstrated by the significant correlations with VCI, PRI, and WMI subscales (Figure 3–5). In parallel, integration properties changes were correlated with FSIQ and VCI in the whole brain networks, and with FSIQ and PRI in the left hemisphere networks. These findings support the association of high intelligence with greater network efficiency throughout the brain, and especially in the left hemisphere. Our results are in agreement with the study of Santarnecchi et al. (2014), that highlighted an association between IQ scores and the GE measures of both strong and weak connections. Moreover, a recent study introduced the idea that differences in intelligence are related to different ways of information processing, with some networks being more efficient in integration and propagation of information across the modules, and others in segregation, i.e., ensuring communication within the module (Hilger et al., 2017b). In line with this hypothesis, our study showed that modifications occurred not only in integration but also in segregation properties, which were correlated with intelligence scores in both whole brain and hemispheres networks. Finally, the correlation found between FC changes of homotopic pairs of regions and FSIQ, as well as VCI and PRI, confirmed that homotopic FC is modified in high intelligence, in agreement with the report of Santarnecchi et al. (2015).

Among all the brain networks differences found in our study, several regions presented decrease or increase in their nodal metrics. It is the case for the dorsolateral prefrontal cortex that showed a significant reduction in D, suggesting less FC. This observation was found both in the whole brain and in the left hemisphere networks of HIQ and Het-HIQ groups. These findings support the hypothesis that this prefrontal region constitutes a weaker node in HIQ children, which might result from a late GM maturation, as previously observed in high intelligence children (Shaw et al., 2006). As the rationale of the hub disruption index κ is to highlight simultaneous decreases in some nodes metrics and increases in others (see Figure 1 for a scheme-based explanation), the D decrease observed in the prefrontal cortex may suggest potential increases of integration properties in other brain areas.

Overall, our study demonstrated the sensitivity of rs-fMRI graph metrics to characterize the specificities in functional brain networks changes of HIQ children, and particularly of Het-HIQ children. As Het-HIQ children could be associated with specific social behavioral and learning difficulties, these findings support our initial hypothesis that FC measurements may constitute a promising approach for a better characterization of HIQ brain function and neural characteristics. Future studies may extend these findings on a larger cohort of children.

### Methodological Limitations

We should underline some limitations related to our dataset. First of all, this study is a pilot study with a low sample size, and needs to be replicated in a larger population. Second, since brain maturation continues during and after childhood, our results may only hold for our particular age range of 8–12 years old. Finally, the population was not equally distributed between girls and boys. Our correlation analysis has, however, been corrected for the effect of gender in order to overcome this problem. From a methodological perspective, this study may have been influenced by the choice of graphs cost. The analysis was thus also computed with a graph cost of 0.20, obtaining concordant results (Supplementary Tables 1, 2).

## Data Availability

The datasets generated for this study are available on request to the corresponding author.

## Ethics Statement

This study was carried out in accordance with the recommendations and approved by the local Ethics Committee (CPP Sud-Est IV) and the French National Agency for Medicine and Health Products Safety (ANSM). Written informed consent was obtained from the parents of all participants and the participants.

## Author Contributions

IS performed all data processing, graph-based analysis, and statistical analysis. CD-M participated in the graph-based analysis. GK participated in the statistical analysis. CS participated in the data acquisition. SH and CS participated in the data processing. SA provided expertise in graph-based analysis. IS, CD-M, GK, CS, SH, AB, and FN wrote the manuscript. PF participated in the study coordination and in interpreting the results. OR participated in the subject inclusion, study coordination, and provided clinical expertise in the interpretation of the results. FN coordinated the study, recruited the children, and provided the clinical expertise in the interpretation of the results. DS-M coordinated the study, supervised MR acquisition, provided methodological MR expertise, participated in the interpretation of results, and supervised the manuscript writing.

## Conflict of Interest Statement

The authors declare that the research was conducted in the absence of any commercial or financial relationships that could be construed as a potential conflict of interest.

## Footnotes

1 https://www.fil.ion.ucl.ac.uk/spm/software/spm12

2 https://www.nitrc.org/projects/conn

3 http://www.brain-connectivity-toolbox.net/

4 http://www.R-project.org/
