# Corrigendum: Topological Modification of Brain Networks Organization in Children With High Intelligence Quotient: A Resting-State fMRI Study

**DOI:** 10.3389/fnhum.2019.00450

**Published:** 2020-01-10

**Authors:** Ilaria Suprano, Chantal Delon-Martin, Gabriel Kocevar, Claudio Stamile, Salem Hannoun, Sophie Achard, Amanpreet Badhwar, Pierre Fourneret, Olivier Revol, Fanny Nusbaum, Dominique Sappey-Marinier

**Affiliations:** ^1^Univ. Lyon, INSA-Lyon, Université Claude Bernard Lyon 1, UJM-Saint Étienne, CNRS, INSERM, CREATIS UMR 5220, Lyon, France; ^2^Univ. Grenoble Alpes, INSERM, U1216, Grenoble Institut Neurosciences, Grenoble, France; ^3^Nehme and Therese Tohme Multiple Sclerosis Center, Faculty of Medicine, American University of Beirut, Beirut, Lebanon; ^4^GIPSA-Lab, UMR CNRS 5216, Université Grenoble Alpes, Grenoble, France; ^5^Centre de Recherche de l'Institut Universitaire de Gériatrie de Montréal, Université de Montréal, Montreal, QC, Canada; ^6^Service de Psychopathologie du Développement de l'Enfant et de l'Adolescent, Hospices Civils de Lyon, Lyon, France; ^7^Laboratoire Parcours Santé Systémique (Equipe d'Accueil 4129), Université de Lyon, Université Claude Bernard-Lyon 1, Lyon, France; ^8^Centre PSYRENE, Lyon, France; ^9^CERMEP – Imagerie du Vivant, Université de Lyon, Lyon, France

**Keywords:** intelligence, functional MRI, resting state, functional connectivity, brain networks, hub disruption index, children

In the original article, there was an error. It was not mentioned in the article that the HIQ group of children included children with either a FSIQ > 130 or a VCI > 130.

A correction has been made to the **Materials and Methods**, subsection **Participants:**

“Fifty-eight children (44 males and 14 females) ages 8–12 (mean age 10.1 ± 1.2) years were recruited from the children psychiatry department of Lyon's Neurological Hospital, the PSYRENE Center, a psychological center for high IQ children and adults, and via advertisement in schools for controls. Children with neurological diseases, learning disabilities, and psychotropic treatments were excluded from this study. Children underwent the fourth edition of WISC (WISC-IV) test and their FSIQ was established from the results of its four subscales (VCI, PRI, WMI, and PSI). Children with a high Intelligence Quotient (FSIQ > 130 or VCI > 130) were labeled as HIQ children and two HIQ profiles were defined based on score difference between VCI and PRI (Table 1). This prospective study was approved by the local ethics committee (CPP Sud-Est IV) and the French National Agency for Medicine and Health Products Safety (ANSM). Written informed consent was obtained from the parents of all participants.”

The authors apologize for this error and state that this does not change the scientific conclusions of the article in any way. The original article has been updated.

