# Supplementary material for: Topological Modification of Brain Networks Organization in Children With High Intelligence Quotient: A Resting-State fMRI Study
Source: Front Hum Neurosci. 2019 Jul 12;13:241. doi: 10.3389/fnhum.2019.00241 (PMC6639736; doi:10.3389/fnhum.2019.00241)
Supplement: Supplementary file 1 [file Table_1.DOCX]

Supplementary Material

**Supplementary Table 1:** Results obtained with a graph cost of 20%: reorganization indices (κ) in Standard Intelligence Quotient (SIQ), High Intelligence Quotient (HIQ), Homogeneous HIQ (Hom-HIQ) and Heterogeneous HIQ (Het-HIQ) groups measured in whole brain networks (A), left and right hemispheres networks (B, C) and between homotopic regions (D).

| **Networks** | ***κ*** | **HIQ** | **Hom-HIQ** | **Het-HIQ** |
| --- | --- | --- | --- | --- |
|  |  | **(n=32)** | **(n=14)** | **(n=18)** |
|  | ***κ_BC_*** | -0.366 | -0.447 | -0.324 |
| **A. Whole** | ***κ_D_*** | -0.280 (**) | -0.285(*) | -0.278 (***) |
| **brain** | ***κ_LE_*** | -0.296 (*) | -0.316 | -0.286 (*) |
|  | ***κ_CC_*** | -0.315 (*) | -0.337 | -0.304 (*) |
|  | ***κ^L^_BC_*** | -0.429 (**) | -0.473 (*) | -0.407 (**) |
| **B. Left** | ***κ^L^_D_*** | -0.256 (**) | -0.304 (*) | -0.231 (**) |
| **hemisphere** | ***κ^L^_LE_*** | -0.221 | -0.213 | -0.225 |
|  | ***κ^L^_CC_*** | -0.285 | -0.288 | -0.283 |
|  | ***κ^R^_BC_*** | -0.232 | -0.212 | -0.242 |
| **C. Right** | ***κ^R^_D_*** | -0.211 | -0.167 | -0.233 (*) |
| **hemisphere** | ***κ^R^_LE_*** | -0.297 | -0.257 | -0.317 (*) |
|  | ***κ^R^_CC_*** | -0.343 | -0.309 | -0.361 |
| **D. Homotopic** | ***κ^HC^*** | -0.185 (**) | -0.261 | -0.145 (*) |

* p<0.05; ** p<0.01; when testing significance of κ values in HIQ, Hom-HIQ or Het-HIQ groups compared to SIQ group using permutation test (number of permutations = 1000)

**Supplementary table 2.** Results obtained with a graph cost of 0.20: coefficients of non-parametric correlations (ρ) between the topological reorganization coefficient (κ) of different nodal metrics (Betweenness Centrality (*BC*), Degree (*D*), Local Efficiency (*LE*), and Clustering (*CC*), and Homotopic Connectivity (*HC*)) with intelligence scores (Full Scale IQ (FSIQ), Verbal Comprehension Index (VCI) and Perceptual Reasoning Index (PRI)) at different network levels: whole brain (A), left and right hemispheres (B and C) and homotopic regions (D).

| **Networks** | ***κ*** | **FSIQ** | **VCI** | **PRI** |
| --- | --- | --- | --- | --- |
|  | ***κ_BC_*** | -0.317 (*) | -0.323 (*) | -0.339 (*) |
| **A. Whole** | ***κ_D_*** | -0.278 (*) | -0.277 (*) | -0.278 (*) |
| **brain** | ***κ_LE_*** | -0.243 | -0.303 (*) | -0.248 |
|  | ***κ_CC_*** | -0.272 | -0.337 (*) | -0.305 (*) |
|  | ***κ^L^_BC_*** | -0.374 (*) | -0.381 (**) | -0.337 (*) |
| **B. Left** | ***κ^L^_D_*** | -0.324 (*) | -0.275 (*) | -0.337 (*) |
| **hemisphere** | ***κ^L^_LE_*** | -0.203 | -0.201 | -0.247 |
|  | ***κ^L^_CC_*** | -0.306 (*) | -0.336 (*) | -0.354 (**) |
|  | ***κ^R^_BC_*** | 0.050 | 0.008 | -0.050 |
| **C. Right** | ***κ^R^_D_*** | -0.215 | -0.252 | -0.163 |
| **hemisphere** | ***κ^R^_LE_*** | -0.092 | -0.196 | -0.087 |
|  | ***κ^R^_CC_*** | -0.166 | -0.264 (*) | -0.170 |
| **D. Homotopic** | ***κ^HC^*** | -0.515 (**) | -0.589 (***) | -0.523 (***) |

* p<0.05; ** p<0.01 when testing significance level of correlations using permutation testing (number of permutations = 1000)
